# The Effect of Exosomes Isolated from Red Blood Cell Concentrates on T Cell Proliferation and Tregs

**DOI:** 10.3390/jcm15197501

**Published:** 2026-09-26

**Authors:** Salih Haldun Bal, Gözde Arslan, Ceren Esen-Kirez, Diğdem Yöyen-Ermiş, Yağmur Aydın-Atalay, Gizem Aytoğu, Kadir Yeşilbağ, Yasemin Heper, Haluk Barbaros Oral

**Affiliations:** 1 Dr. Raşit DURUSOY Blood Bank, Faculty of Medicine, Bursa Uludag University, 16059 Bursa, Türkiye; yheper@uludag.edu.tr; 2 Department of Immunology, Faculty of Medicine, Bursa Uludag University, 16059 Bursa, Türkiye; arslangozde9337@gmail.com (G.A.); ccereneesen@gmail.com (C.E.-K.); dyoyenermis@uludag.edu.tr (D.Y.-E.); aydinyagmurbio@gmail.com (Y.A.-A.); oralb@uludag.edu.tr (H.B.O.); 3 Experimental Animal Breeding and Research Unit, Faculty of Medicine, Bursa Uludag University, 16059 Bursa, Türkiye; 4 Experimental and Molecular Immunology Laboratory (Experim), Bursa Uludag University, 16059 Bursa, Türkiye; 5 Department of Medicine-Immunology, Institute of Health Science, Bursa Uludağ University, 16059 Bursa, Türkiye; 6 Department of Virology, Faculty of Veterinary Medicine, Bursa Uludag University, 16059 Bursa, Türkiye; gizemalpay@uludag.edu.tr (G.A.); kyesilbag@uludag.edu.tr (K.Y.); 7 Department of Infectious Diseases and Clinical Microbiology, Faculty of Medicine, Bursa Uludag University, 16059 Bursa, Türkiye

**Keywords:** exosomes, leukoreduction, red blood cell concentrate, regulatory T cells, T cell proliferation, transfusion-related immunomodulation

## Abstract

**Background/Objectives**: Transfusion-Related Immunomodulation (TRIM) is a complex biological phenomenon that develops following allogeneic blood transfusions. Exosomes released from stored red blood cell concentrates (RBC) may be a potential mediator of TRIM. This study aims to analyze the effects of exosomes on T cell proliferation and regulatory T cell (Treg) differentiation. **Methods**: RBC concentrates obtained from six healthy donors were divided into non-leukoreduced (NR) and leukoreduced (LR) units. Exosomes were isolated from these RBCs by ultracentrifugation. Allogeneic and autologous exosome pools were prepared. Peripheral blood mononuclear cells (PBMCs) were cultured with exosome pools (with 0.2, 1, or 5 μg doses) under allogeneic or autologous conditions. T-cell proliferation and Tregs were assessed by flow cytometry. **Results**: Total exosomal protein content increased during storage, with significantly higher levels in NR-RBC derived exosomes (NR-Exo) than LR-RBC derived exosomes (LR-Exo). NR-Exos significantly enhanced both CD4^+^ and CD8^+^ T cell proliferation, predominantly in allogeneic cultures. The proliferative effect was observed at different doses. In contrast, exosome treatment did not promote expansion of Treg populations. Instead, autologous cultures demonstrated reduced frequencies of CD8^+^ Tregs and CD39-expressing Tregs, whereas allogeneic cultures showed minimal changes. Moreover, leukoreduction attenuated exosome-mediated T-cell proliferation. **Conclusions**: RBC-Exos preferentially enhance effector T cell proliferation under allogeneic conditions, particularly when isolated from NR-RBC units. Tregs tended to decrease in both the autologous and leukoreduced groups. These findings suggest that RBC-Exos support both immunosuppressive effects and immune activation. However, the characteristics of the RBC and the transfusion (allogeneic/autologous and leukoreduced/non-leukoreduced) are considered the key factors in observing these effects.

## 1. Introduction

Transfusion-associated immunomodulation (TRIM) is a transfusion reaction that describes changes in the function of the immune system that occur in the recipient following allogeneic blood transfusion (ABT) [1,2]. TRIM was first defined in 1973, based on observations of the positive effects of transfusions on graft survival in patients awaiting kidney transplants [3]. It has subsequently been reported that tumor growth and metastases are increased in patients who receive ABT prior to curative surgery [4]. After the adverse effects of ABT were noticed, the effects of transfusion on the recipient’s immune system became an area of interest. Several studies have reported that ABT reduces the frequency of spontaneous miscarriage and Crohn’s disease relapse [5,6]; however, it increases the frequency of postoperative bacterial infections, the likelihood of reactivation of some latent infections such as cytomegalovirus (CMV), and the risk of all-cause mortality [7,8,9,10].

It has also been suggested that ABT causes these clinical conditions through mechanisms such as decreased helper T (Th) cell number, decreased T cell response, Treg activation, decreased natural killer cell function, decreased CD4^+^/CD8^+^ ratio, inadequate antigen presentation, and decreased monocyte/macrophage phagocytic function [11,12,13]. It is thought that ABT may produce this effect through mononuclear cells (MNC) in the blood component, biological response modifiers and immunological mediators (BRM-IM) that accumulate in the blood component supernatant, and soluble human leukocyte antigen (HLA) class I peptides in allogeneic plasma [1,2]. Therefore, several studies have reported that removing allogeneic MNCs from the blood component (leukoreduction) is an effective strategy to prevent TRIM development [14,15,16,17]. However, some studies have indicated that leukoreduction is an insufficient method to prevent TRIM [18,19,20,21]. Contradictions in the literature and the possibility that allogeneic leukocytes are being eliminated by the recipient’s immune system suggest that BRM-IMs may play a more prominent role in TRIM development. It has been reported that a wide variety of BRM-IMs—including cytokines, eicosanoids, growth factors, ubiquitin, and tissue plasminogen activator—contribute to immunomodulation and support Treg development [13,22]. Exosomes that accumulate within blood components during the storage time also have effects on the recipient’s immune system. It has been shown that exosomes isolated from red blood cell concentrate (RBC) enhance the activity of antigen-presenting cells and strengthen T-cell proliferative responses [23].

This study aims to evaluate the effects of exosomes isolated from RBCs on T cell proliferation and Treg frequency in in vitro cell cultures, thereby providing insights into their roles in Treg development. In this study, the effects of exosomes isolated from leukoreduced RBCs (LR-Exo) and exosomes isolated from non-leukoreduced RBCs (NR-Exo) were compared to analyze the effect of leukoreduction. Additionally, both allogeneic and autologous cultures were established using LR-Exo and NR-Exo to investigate the role of allogeneicity in the observed effects, as well as the impact of exosome dose.

## 2. Materials and Methods

Briefly, LR-RBC and NR-RBC samples were first prepared, and then exosomes were isolated from these samples. To characterize the exosomes, exosomal proteins were detected by Western blot (WB), size and concentration were analyzed using Tunable Resistive Pulse Sensing (TRPS), and tetraspanins were evaluated by flow cytometry (FC) [24]. After characterization, it was confirmed that the isolates were exosomes, and functional experiments were then conducted to investigate the effects of exosomes on T cell proliferation and Treg frequency.

### 2.1. Preparation of LR-RBC and NR-RBC Samples

Six units of whole blood were collected from six blood donors (Reveos Automated Blood Processing System, Terumo, Lakewood, CO, USA). Each whole blood was separated into its components using a fully automated blood processing system (Reveos, Terumo). The RBCs were then divided into two equal parts, and log4 leukoreduction via filtration was applied to one of these parts. Thus, LR-RBCs and NR-RBCs were obtained from the same donation by the same donor (Figure 1a). The LR-RBCs and NR-RBCs were then divided into three equal parts to provide samples for storage days 0, 21, and 42 (NR-RBC-D0, NR-RBC-D21, NR-RBC-D42; LR-RBC-D0, LR-RBC-D21, LR-RBC-D42). These samples were stored at +4 °C in blood storage refrigerators (Angelantoni Life Science, PG, Italy) for 42 days (Figure 1). Exosomes were isolated immediately from D0 samples, while they were isolated from D21 and D42 samples on the respective days.

### 2.2. Isolation of Exosomes from LR-RBC and NR-RBC Samples

Isolations were performed at +4 °C, while maintaining sterility, through sequential centrifugations. The supernatants of the LR-RBC and NR-RBC units were separated by centrifugation at 300× *g* for 10 min (Nüve, NF 800R, Ankara, Türkiye). The separated supernatants were first centrifuged at 3000× *g* for 10 min, and the supernatant was then ultracentrifuged (Optima XPN-100, Beckman-Coulter, Indianapolis, IN, USA) at 15,000× *g* for 35 min (Figure 1). The pellet was discarded, and the supernatant was then filtered through a 0.22 µm filter (cat#99722, TPP Techno Plastic Products AG, Trasandingen, Switzerland). In the next step, exosomes were isolated from the filtered supernatant by ultracentrifugation. The supernatants were centrifuged in an ultracentrifuge using a SW 41Ti swing rotor (Beckman-Coulter) at 125,000× *g* for 2 h (Figure 1a). Following this step, a washing step was performed by centrifuging at 125,000× *g* for 60 min. After washing, the exosome pellets were resuspended in exosome-free PBS (efPBS) to obtain the NR-D0-Exo, NR-D21-Exo, NR-D42-Exo, LR-D0-Exo, LR-D21-Exo, and LR-D42-Exo samples. The samples were stored at −80 °C (DW-HL528, Meling Biology & Medical, Hefei, China) (Figure 1a).

### 2.3. Measurement of Exosomal Protein Levels

Exosomal protein levels were analyzed in all NR-D0-Exo, NR-D21-Exo, NR-D42-Exo, LR-D0-Exo, LR-D21-Exo, and LR-D42-Exo samples using the MicroBCA Assay (Pierce, Thermo Scientific, Rockford, IL, USA). Each exosome sample was tested in triplicate, and the analysis was performed in accordance with the manufacturer’s instructions. The results were calculated using optical density values obtained by reading the plates on a spectrophotometer (BioTek uquant MXQ200 Microplate Reader, Illinois, IL, USA).

### 2.4. Western Blot (WB) Analysis of Exosomal Proteins

Exosome samples were pooled at equal concentrations to achieve a final concentration of 1500 μg/mL. The pool was then lysed using RIPA buffer at +4 °C, 15,000× *g* for 15 min to extract exosomal proteins. It was mixed with loading buffer and incubated at 95 °C for 5 min. Subsequently, SDS-PAGE was prepared by using a Protein Ladder (cat#926-98000, LI-COR, Lincoln, NE, USA), Running Buffer, and a running unit (Clever Scientific, Warwickshire, UK). The transfer module was covered with filter paper, a PVDF membrane, the SDS-PAGE gel, and transfer paper, and placed in a Semi-Blot transfer device (Clever Science, Warwickshire, UK). The PVDF membrane was blocked in TBS-T (Tris-buffered saline-Tween 20) Western blot washing solution. Next, the exosomal proteins were analyzed using the Rabbit-produced antibodies. The anti-TSG101 antibody (cat#72312S, Cell Signaling Technology, Danvers, MA, USA), the anti-Alix antibody (cat#92880S, Cell Signaling Technology, Danvers, MA, USA), the anti-CD9 antibody (cat#13174S, Cell Signaling Technology, USA), the anti-CD63 antibody (cat#52090S, Cell Signaling Technology, USA), the anti-CD81 (cat#56039S, Cell Signaling Technology, USA) and PVDF membrane were incubated overnight in the refrigerator [25]. In addition, the Rabbit-Calnexin antibody (cat#2679S, Cell Signaling Technology, USA), which is specific to calnexin, a protein not found in exosomes or present only in very low concentrations, was also used [26]. The next day, the membrane was incubated with the Secondary Rabbit-IgG-HRP antibody (cat#7074S, Cell Signaling Technology, USA) and imaged using an imaging system (C-Digit, Li-Cor, Nebraska, NE, USA) with ECL substrate (cat#20-500-120, ECL EZ kit, Biological Industries, Beit-Haemek, Israel).

### 2.5. Tunable Resistive Pulse Sensing (TRPS)

This analysis was performed on a randomly selected sample by using the qNano Exoid (Izon Biosciences, Christchurch, New Zealand) instrument and the Exoid Control Suite (Izon Biosciences, New Zealand) software. The NP150 nanopore (Izon Biosciences, New Zealand) was used. After suspending the exosome samples (1×) in efPBS, the analysis was performed using 40 µL of the exosome sample. The readout process was initiated with reference values of 47 mm nanopore tension, 700 mBar pressure, and 0.47 mV voltage, and necessary adjustments were made based on the readout conditions. The reading process was continued until 500 particles were counted. Since the values obtained during the reading process were expressed in nA for size and in particles per minute for concentration, the actual size and concentration values were determined by reading the CPC100 carboxylated polystyrene calibration beads (Izon Biosciences, New Zealand) [27].

### 2.6. Tetraspanin Analysis

To ensure the visibility of exosomes in FC, carboxylate beads (3.5 µm, Invitrogen, Carlsbad, CA, USA) were first coated with the purified monoclonal anti-CD9 Ab (cat#555370, BD Bioscience, San Jose, CA, USA). Then, the exosomes were conjugated to these beads. To coat the beads, 50 µL of beads was incubated with 10 μg of purified monoclonal anti-CD9 Ab (BD Bioscience) at room temperature, then the volume was adjusted to 500 µL with efPBS. After overnight incubation at room temperature, the mixture was centrifuged, the supernatant was discarded, and 5% bovine serum albumin (BSA) was added. After four hours of incubation, the supernatant was discarded, and the beads were resuspended in 1% BSA to obtain anti-CD9 antibody-coated beads. To conjugate the exosomes with these anti-CD9 antibody-coated beads, 1 µL of antibody-coated beads was used for every 1 µg of exosomes. Exosomes and beads were incubated overnight at room temperature. For FC analysis, 1 µg of exosome/bead conjugate was used for each staining. The exosome/bead conjugate was then labeled with anti-CD9 APC (cat#312108, BioLegend; San Diego, CA, USA), anti-CD63 FITC (cat#MA1-19602, Invitrogen, USA), and anti-CD81 PE (cat#349506, BioLegend; San Diego, CA, USA). The samples were incubated for 1 h at room temperature in the dark. Then, samples were washed with 500 µL of efPBS to remove unbound antibodies and resuspended in 300 µL of efPBS. Expression was analyzed by flow-cytometry (FC) (FACSymphony A1, BD Bioscience) [27].

### 2.7. Preparation of Exosome Pools

For functional analyses, allogeneic and autologous exosome pools were prepared. Two large allogeneic exosome pools (LR Pool and NR Pool) were prepared by mixing equal concentrations of samples from NR-D0-Exo, NR-D21-Exo, NR-D42-Exo, and LR-D0-Exo, LR-D21-Exo, LR-D42-Exo (Figure 1b). For the autologous exosome pools, the D0, D21, and D42 exosomes from each donor were pooled at equal concentrations (LR Individual Pool and NR Individual Pool) (Figure 1c). These exosome pools were used in functional experiments.

### 2.8. Preparation of Allogeneic and Autologous PBMC Cultures with Exosome Pools

Blood samples were obtained from volunteers for allogeneic cultures and from individuals who donated RBCs for autologous cultures. Six volunteers were used to proliferation, and five volunteers were used for Treg experiments. PBMCs were isolated using the density gradient method (Ficoll-1.077, Capricorn Scientific, Germany) from the blood samples. The number of PBMCs was counted via the Thoma slide by using trypan blue, and the quantities of cells to be used in the culture wells were calculated.

To evaluate the effects of the LR Pool, NR Pool, LR Individual Pool, and NR Individual Pool on T cell proliferation, PBMCs were used from six volunteers and five blood donors. Three wells were allocated for each condition. After the cells were diluted in 1 mL of RPMI 1640, CFSE was added at a concentration of 1 µL per 1,000,000 cells (1 µM), and the mixture was incubated at 37 °C for 15 min. Then, 7 mL of complete RPMI 1640 (cRPMI) was added, and the mixture was allowed to stand for 5 min. The sample was centrifuged at 4 °C for 5 min, and after removing the supernatant, the same procedure was repeated. The cell pellet was resuspended in 200 µL of exosome-free complete RPMI 1640 (efcRPMI) to a concentration of 100,000 cells. All procedures were performed in the dark. The cells (100,000 PBMC/200 µL efcRPMI) were added to a U-bottom 96-well culture plate. To induce proliferation 1 µL (1 µM) phytohemagglutinin (PHA; Merck, Darmstadt, Germany) was administered. Then, exosomes were added to the wells from the LR Pool, NR Pool, LR Individual Pool, or NR Individual Pool at doses of 0.2 µg, 1 µg, or 5 µg (1×, 5×, and 25×). LR Pool and NR Pool samples were added to allogeneic PBMCs (Figure 1b), while LR Individual and NR Individual samples were added to autologous PBMCs (Figure 1c). Since the exosome samples were resuspended in efPBS, efPBS was used to form the control group. At the end of the 96-h incubation, T-cell proliferation was assessed by (Navios EX, Beckman Coulter) using anti-CD3 APC (cat#A94680, Beckman Coulter, Indianapolis, IN, USA) and anti-CD8 PE (cat#A94680, Beckman Couler) monoclonal antibodies. The cells collected from the wells were washed with 500 μL of efPBS, then labeled with CD3 and CD8 monoclonal antibodies and incubated at room temperature for 25 min. At the end of the incubation, the cells were washed again with 500 μL of efPBS. The cells were resuspended in 300 μL of efPBS and analyzed.

To evaluate the effects of the LR Pool, NR Pool, LR Individual Pool, and NR Individual Pool on Treg development, five allogeneic and five autologous PBMC samples were used. Each condition was tested in triplicate. After adding 100,000 PBMCs per well, 1 µL (1 µM) of PHA (Merck) was added to the wells. Exosome samples were then added to the wells at doses of 0.2 µg, 1 µg, and 5 µg (1x, 5x, and 25x). Only efPBS was added to the control well. After 96 h of incubation, the wells were harvested separately and washed with efPBS. For autofluorescence analysis, unstained fractions were used. For FC analysis, anti-CD3-APC AF750 (cat#A94680, Beckman Coulter), anti-CD4 ECD (cat# 6604727, Beckman Coulter), anti-CD8 APC-AF700 (cat#B49181, Beckman Coulter), anti-CD25-PE (cat#A07774, Beckman Coulter), anti-CD39-PC5.5 (cat#B555385, Beckman Coulter), and anti-CD127 APC (cat#B42026, Beckman Coulter) monoclonal antibodies were used for surface staining. Intracellular staining kit (PerFix-nc; cat#B31168, Beckman Coulter) and anti-FoxP3 PC7 (cat#B46032, Beckman Coulter) monoclonal antibody were used for that purpose. Before the assay, a monoclonal antibody mixture was prepared for surface staining, and a 15 µL volume of this mixture was added to 60 µL of PBMCs. Following a 15-min incubation at room temperature in the dark, 5 µL of Fixative Reagent (PerFix-nc) was added, and the incubation was repeated under the same conditions. After adding 300 µL of Permeabilizing Reagent (PerFix-nc) and anti-FoxP3 PC7, the sample was incubated for 60 min at room temperature in the dark. Following two washes, the pellet was collected and analyzed by FC (Navios EX, Beckman Coulter). In addition to the phenotypic assessment of Tregs, the anti-CD39 monoclonal antibody was added to the panel to evaluate their functional immunosuppressive potential [28,29].

### 2.9. Statistical Analysis

Descriptive statistics for continuous variables are presented as the median (min–max). Descriptive statistics for categorical data are reported as frequency and percentage. The normality of the data distribution was assessed using the Shapiro–Wilk test. To compare the two groups, nonparametric tests—including the Mann–Whitney U test and the Wilcoxon test—and parametric tests—including the paired *t*-test and the unpaired *t*-test—were used. Data analysis was performed using GraphPad Prism 9 software. The significance level was set at α = 0.05. *p*-values ranging from 0.050 to 0.065 were described as trends or limited significance.

## 3. Results

### 3.1. Exosomal Protein Levels

In the NR-Exo group, a significant increase in total exosomal protein levels was observed, related to storage time. The protein concentrations in the D21 and D42 samples were found to be significantly higher than those in the D0 samples (*p* < 0.01). Although protein levels were higher in the D42 group compared to D21, no statistical significance was detected between these two time points. In the LR-Exo group, a more limited increase in protein levels was observed in relation to storage time. While the total exosomal protein concentration in D42 samples was significantly higher than that in D0 samples (*p* < 0.05), no significant difference was observed between D21 and the other time points. In between-group comparisons, the total exosomal protein level in the NR-Exo group was found to be significantly higher than that in the LR-Exo group in the D21 samples (*p* < 0.05). In contrast, no statistically significant difference was detected between the NR-Exo and LR-Exo groups at the D0 and D42 time points (Figure 2a).

### 3.2. Western Blot Analysis of Exosomal Proteins

TSG101 and CD9 bands were detected in WB analyses, while Alix, CD63, and CD81 bands were not seen. RAW lysate and the THP-1 cell line were used as negative controls. The calnexin band was detected in the RAW lysate and the THP-1 cell line, while the band did not occur in the exosome samples (Figure 2b). The presence of the TSG101 and CD9 bands and the absence of the calnexin band were accepted as evidence [24].

### 3.3. Tunable Resistive Pulse Sensing (TRPS)

Based on three consecutive measurements, the average diameters of the particles were determined to be 100–117 nM; their mode diameters, 87–103 nM; and their concentrations, 1.02 × 10^10^ particles/mL (Figure 2c). The determined particle diameters were found to be consistent with the reported exosome size [24,25].

### 3.4. Analysis of Tetraspanins by Flow Cytometry

While CD63 frequencies were not detected in exosome samples, CD9 and CD81 were detected (Figure 2d). This result was considered sufficient to conclude that the samples were exosomes [24].

### 3.5. The Effects of Exosomes on T Cell Proliferation

The effects of LR Pool, NR Pool, LR Individual Pool, and NR Individual Pool exosomes on the proliferation of CD3^+^CD4^+^ and CD3^+^CD8^+^ T cells in allogeneic and autologous PBMC cultures were evaluated by FC (Figure 3). The results were normalized with reference to the control group. Statistical analyses were performed according to the control group, the doses, and between the groups.

The results of the allogeneic culture (Allo), the autologous culture (Auto), the NR culture, and the LR culture were evaluated from a general perspective. CD3^+^CD4^+^ T cell proliferation showed a significant increase compared to the control group in the Allo group (*p* < 0.01) and in the NR group (*p* < 0.05) (Figure 3a). Additionally, CD3^+^CD4^+^ T cell proliferation was found to be significantly higher in the NR group compared to the LR group (*p* < 0.05) (Figure 3a). In the evaluation of the effect of leukoreduction in allogeneic and autologous cultures, the proliferation in the Allo-NR group was found to be significantly higher than in the control group (*p* < 0.05) (Figure 3b). However, it was found that the proliferation of CD3^+^CD4^+^ cells in the Auto-NR group tended to be higher than in the Auto-LR group (*p* = 0.062) (Figure 3b). The effect of exosome doses was evaluated. CD3^+^CD4^+^ T cell proliferation at a 5 µg exosome dose was found to be significantly higher in the NR Pool group compared to the LR Pool group in allogeneic cultures (*p* < 0.05) (Figure 3c). No significant differences were observed among the other doses or in autologous cultures (Figure 3c,d).

In the overall analysis of CD3^+^CD8^+^ T cell proliferation, a significant increase in proliferation was observed in the Allo and NR groups compared to the control group (*p* < 0.001) (Figure 3e). Additionally, proliferation was found to be significantly higher in the NR group compared to the LR group (*p* < 0.05) (Figure 3e). In the evaluation of the effect of leukoreduction in allogeneic and autologous cultures, CD3^+^CD8^+^ proliferation was found to be significantly higher in the Allo-NR group compared to the control group (*p* < 0.01) and significantly higher compared to the Allo-LR group (*p* < 0.05). Also, proliferation showed an increasing trend in the Allo-NR group compared to the Auto-NR group (*p* = 0.053) (Figure 3f). When the effect of exosome dose was evaluated, CD3^+^CD8^+^ T cell proliferation in allogeneic cultures was found to be significantly higher in the NR Pool group compared to the control group at a 0.2 µg exosome dose and compared to the LR Pool group at a 5 µg exosome dose (*p* < 0.05) (Figure 3g). No significant differences were observed among the other doses or in autologous cultures (Figure 3g,h).

### 3.6. The Effects of Exosomes on Tregs

The effects of LR Pool, NR Pool, LR Individual Pool, and NR Individual Pool exosomes on the development of CD3^+^CD4^+^ and CD3^+^CD8^+^ Treg cells in allogeneic and autologous PBMC cultures were evaluated by FC. The results were normalized with reference to the control group. Statistical analyses were performed according to the control group, the doses, and between the groups (Figure 4).

In the overall analysis conducted according to the main groups, Allo, Auto, NR, and LR, a tendency toward a decrease in CD4^+^ Treg (CD3^+^CD4^+^CD25^High^CD127^Low^FoxP3^High^) cells was observed in the Auto group compared to the control group (*p* = 0.064) (Figure 4a). In the evaluation of the effect of leukoreduction in allogeneic and autologous cultures, a tendency toward a decrease in CD4^+^ Treg cells in the Auto-LR group compared to the Auto-NR group (*p* = 0.065) (Figure 4b). Compared to the control group, CD8^+^ Treg (CD3^+^CD8^+^CD25^High^CD127^Low^FoxP3^High^) cells were found to be significantly lower (*p* < 0.05) in the Auto group, and showed a decreasing tendency in the LR group (*p* = 0.064) (Figure 4c). When the effect of leukoreduction on CD8^+^ Treg cells was evaluated in allogeneic and autologous cultures, no significant change was observed (Figure 4d).

When CD39 expressing CD4^+^ Treg cells were examined (CD3^+^CD4^+^CD39^+^CD25^High^ CD127^Low^FoxP3^High^), their frequencies tended to decrease in the Auto group compared to the control group (*p* = 0.058) (Figure 4e). In the evaluation of the effect of leukoreduction in allogeneic and autologous cultures, CD39 expressing CD4^+^ Tregs were found to be lower in the Auto-LR group compared to the control group, but not significantly (*p* = 0.057) (Figure 4f). When CD39 expressing CD8^+^ Treg cells were examined (CD3^+^CD8^+^CD39^+^CD25^High^CD127^Low^FoxP3^High^), their frequencies were found to be significantly lower in the Auto group compared to the control group (*p* < 0.05) (Figure 4g). When the effect of leukoreduction on CD8^+^ Treg cells was evaluated in allogeneic and autologous cultures, no significant change was observed (Figure 4h).

In our study, we also examined the effects of different doses (0.2, 1, and 5 µg) of exosomes of NR Pool, LR Pool, NR Individual Pool, and LR Individual Pool on Tregs in allogeneic and autologous PBMC cultures (Figure 5). While there were no significant differences among the doses in allogeneic cultures for CD4^+^ Tregs, the LR Individual group in autologous cultures showed a decrease at the 5 µg dose compared to the control group (*p* < 0.05) (Figure 5a,b). Similarly, no significant differences were observed between doses in allogeneic and autologous cultures for CD8^+^ Tregs. However, a trend toward a decrease was observed in the 1 µg dose of the LR Individual group in autologous cultures compared to the control group (*p* = 0.063) (Figure 5c,d).

CD39 expressing CD4^+^ Tregs were found to be significantly higher in the 0.2 µg dose of the NR Pool group in allogeneic cultures compared to the 5 µg dose (*p* < 0.05) (Figure 5e). In autologous cultures, however, the 5 µg dose in the LR Individual group showed a trend toward a decrease compared to the control group (*p* = 0.063) (Figure 5f). CD39 expressing CD8^+^ Tregs, however, showed no significant differences between exosome doses or NR-LR groups in either allogeneic or autologous cultures (*p* > 0.05) (Figure 5g,h).

## 4. Discussion

To date, our team has focused on T lymphocytes within RBCs, evaluating T cell polarization, proliferation, viability, CD8^+^ T cell subsets, and the CD4^+^/CD8^+^ ratio within blood bags [30,31,32,33]. This study is the first investigation of the immunomodulatory effects of exosomes in RBC supernatant.

It is known that extracellular vesicles (EVs) accumulate in RBCs during storage, that exosomes among them exhibit highly heterogeneous biological properties, and that they can interact with immune cells through various mechanisms [34]. In parallel, our study found that exosomal protein levels were elevated in both the NR-Exo and LR-Exo groups. These findings may suggest that exosome release persists throughout the storage period in both groups. It is known that exosome expression levels in cells vary in response to various stimuli [25]. However, red blood cells do not express exosomes [35,36]. The significantly higher levels of exosomal proteins, particularly in the NR-Exo group, may be due to the expression of these proteins by leukocytes in the product as a result of the storage conditions (+4 °C). Interestingly, the increase in protein levels in LR-Exo samples is noteworthy. The fact that platelet and leukocyte-derived EVs have been reported in much lower concentrations in LR-RBCs compared to NR-RBCs makes the increase in LR-Exo samples even more intriguing [37]. However, cells can also secrete exosomes when they undergo apoptosis [38]. In both groups, the increase toward the end of the storage time may have been caused by apoptotic cells. The difference between the groups will result in different effects on the recipient following transfusion. Therefore, in our in vitro study which aimed to simulate the exosome-mediated effect of transfusion, the LR Pool, NR Pool, LR Individual Pool, and NR Individual Pool were prepared by taking equal concentrations from the NR-D0-Exo, NR-D21-Exo, NR-D42-Exo, LR-D0-Exo, LR-D21 -Exo, and LR-D42-Exo samples.

### 4.1. Effects of RBC-Derived Exosomes on T-Cell Proliferation

Danesh and colleagues demonstrated that exosomes isolated from stored RBCs significantly increased the proliferation of CD3^+^CD4^+^ and CD3^+^CD8^+^ T cells [23]. In our study, CD3^+^CD4^+^ and CD3^+^CD8^+^ T cell proliferation were significantly increased in the allogeneic, the NR, and the Allo-NR group. This increase we observed is consistent with the findings of Danesh and colleagues [23]. In addition, Danesh and colleagues have shown that the effect of exosomes on T cell proliferation occurs through the activation of monocytes and antigen-presenting cells. This finding has been interpreted as the primary target of RBC exosomes is antigen-presenting cells, not T cells [23]. This suggests that the results obtained in our study, conducted using PBMC cultures, may also have arisen through a similar mechanism. In our study, the fact that CD3^+^CD4^+^ and CD3^+^CD8^+^ T cell proliferation increased predominantly in allogeneic cultures, while autologous cultures remained largely unaffected, supports this view. Exosomes that accumulate within RBCs during storage and originate from other cells or cell residues in the RBCs can carry HLA molecules. Unlike in autologous cultures, these molecules may have stimulated T cells in allogeneic cultures through antigen-presenting cells. However, our study does not include an experiment to validate this hypothesis. Verifying this hypothesis has become one of our primary goals for the future.

In our study, the significant increase in T cell proliferation, particularly in the NR and Allo-NR groups, suggests that residual leukocytes may have altered the exosomal cargo during storage. This is because the cargo depends on the parent cell’s physiological state at that moment [39]. Furthermore, the size, concentration, composition, and origin of exosomes may vary depending on the method used in the preparation of RBCs [40,41,42]. It has also been reported in the literature that leukoreduction alters the composition of the vesicles [43]. Consistent with this, our study found that the NR group stimulated T cell proliferation more potently than the LR group, and that this effect was most pronounced in the high dose (5 µg) NR Pool group. This is seen as similar to the literature, indicating that leukoreduction can not prevent immunomodulatory effects of ABT [18,19,20,21].

Another important point is that our proliferation results were associated with increased exosomal protein levels. The fact that the 5 µg dose of NR Pool induced a higher proliferative response than LR Pool in allogeneic cultures suggests that the immunomodulatory effect may be related to exosome levels. However, given that RBC storage time and isolation method affect exosome content, and that different protein and miRNA loads can alter cellular signaling networks in a dose-dependent manner, it is clear that the composition of exosomes may be just as important as their load [34,44]. Proteomic studies conducted in recent years have shown that leukoreduction significantly alters the protein and miRNA contents of RBC-derived EVs [45,46]. The effect we observed may also be influenced by the exosome cargo, regardless of the dose. However, our study does not include proteomic and genomic analyses to support this view.

The literature also indicates that T cell proliferation can be suppressed during the TRIM process and that TRIM development can be reduced through leukoreduction [14,15,16,17]. Our results appear to contradict the literature. However, the group of cells whose proliferation was suppressed in the LR groups may also include Tregs. Thus, leukoreduction may play a preventive role in the development of TRIM by reducing Treg frequency. To test this hypothesis, we evaluated the effects of the NR-Exo and LR-Exo groups on CD3^+^CD4^+^ and CD3^+^CD8^+^ Treg cells in allogeneic and autologous environments in a subsequent part of our study.

### 4.2. Effects of RBC-Derived Exosomes on Treg Frequency

It has been suggested that ABT stimulates Tregs, increases the production of immunosuppressive cytokines, and suppresses effector T-cell responses [1,13,34]. Baumgartner and colleagues reported that RBC supernatant increases the number and function of FoxP3^+^ Treg cells [13]. However, studies investigating the effects of RBC-derived exosomes on Treg biology are quite limited. Therefore, in our study, we assessed the effects of exosomes isolated from RBC supernatants on CD3^+^CD4^+^ and CD3^+^CD8^+^ Tregs. Contrary to their proliferative effects, and in contrast to Baumgartner’s study, RBC-derived exosomes tended to reduce Treg frequencies under certain conditions. This may have arisen from methodological differences. Whilst Baumgartner’s study was performed with RBC supernatants, our study used only the exosomes. This situation may be due to the supernatant’s content, as the supernatant contains a large amount of cellular debris and various BRM-IMs.

The significant reduction in CD3^+^CD8^+^ and CD3^+^CD8^+^CD39^+^ Treg frequencies in autologous cultures compared with the control group, and the limited significant reduction in CD3^+^CD4^+^ and CD3^+^CD4^+^CD39^+^ Treg levels, suggest that exosomes may affect the Tregs. CD39, a functional marker of Tregs, represents the suppressive capacity of these cells [28,29]. The fact that frequencies of CD39^+^ Tregs follow a similar pattern to those of other Tregs indicates that the suppressive capacity of Tregs is also reduced. Furthermore, the observation that the effect on CD3^+^CD4^+^ and CD3^+^CD4^+^CD39^+^ Tregs is particularly evident in autologous cultures within the Auto-LR group may highlight the importance of allogenicity in TRIM development and the inhibitory role of leukoreduction. The observed decrease in CD4^+^ Treg frequencies in the Auto-LR group compared to the Auto-NR group, and in CD8^+^ Treg and CD39^+^CD4^+^ Treg levels in the Auto-LR group compared to the control group, appears to be due to cargo changes resulting from leukoreduction [45,46]. However, the lack of a significant difference between the LR and NR groups suggests that allogeneity and the leukocyte content of RBCs play a combined role in Treg frequency. Consequently, leukoreduced autologous RBCs appear to slow down immunomodulation by suppressing Treg frequency. It is also important to bear in mind that immunomodulation cannot be explained solely by an increase in the number of Tregs, but may also occur through changes in Treg function [47,48]. In our study, Tregs expressing CD39 showed results consistent with those of other Tregs. This finding suggests that not only the number of Tregs but also their suppressive function has decreased.

When comparing our proliferation and Treg results, parallel trends are observed. In the Allo and NR groups, both cell proliferation and Treg levels appear to be higher compared to the Auto and LR groups. While the increase in proliferation was significant in the Allo and NR groups, the decrease in Tregs in the Auto and LR groups was found to be significant. It is possible to consider that HLA is one of the key differences between the Allo and NR groups and the Auto and LR groups. This finding suggests that foreign HLA molecules carried by exosomes may play a pivotal role in T cell and Treg proliferation. For this reason, foreign HLA molecules carried by exosomes may be the source of the proposed association between TRIM and soluble HLA molecules found in RBC supernatant [49]. This foresight is among our goals for the future.

The lower Treg frequencies may also be attributable to the experimental design. As is well known, Tregs are regulatory cells and play a role in immune regulation. For this reason, they are activated later than other effector cells. It has been reported that incubation of 96–120 h may be required for stable FoxP3 expression and the development of functional Tregs in cell cultures [50]. The 96-h incubation period used in our study appears to be sufficient. Nevertheless, additional experiments lasting 120 h could have been conducted, but this was not possible due to resource constraints. In addition, our decision not to use mediators such as IL-2 that induce FoxP3 expression, to assess the pure effect of exosomes, may also have contributed to this result [51].

There are also some limitations in our study. While the proliferative capacity was examined, cytokine secretion and T cell activation markers could not be analyzed. Tregs were evaluated only phenotypically; functional suppression assays were not performed, and cytokines such as IL-2, IL-10, and TGF-β were not measured. Due to budget constraints, the sample size could not be expanded, and no proteomic or transcriptomic profiling was performed.

## 5. Conclusions

Our study demonstrates that RBC derived exosomes may play a role in TRIM. In the allogeneic and non-leukoreduced groups, this effect may be immunostimulatory, whereas in the autologous and leukoreduced groups, it may be immunosuppressive. This finding appears consistent with the current view, suggesting that RBC derived exosomes support both immunosuppressive effects and immune activation [34]. However, the characteristics of the RBCs and the transfusion type (allogeneic/autologous and leukoreduced/non-leukoreduced) appear to be the key factors in the observation of these effects. Furthermore, investigating the effect of exosomal HLAs on T cell proliferation and Tregs will be one of our priorities in the near future. Additionally, future research incorporating proteomic, transcriptomic, and functional assays will be valuable for identifying the responsible molecular pathways and elucidating the contribution of RBC exosomes to TRIM pathogenesis.

## Figures and Tables

**Figure 1:**
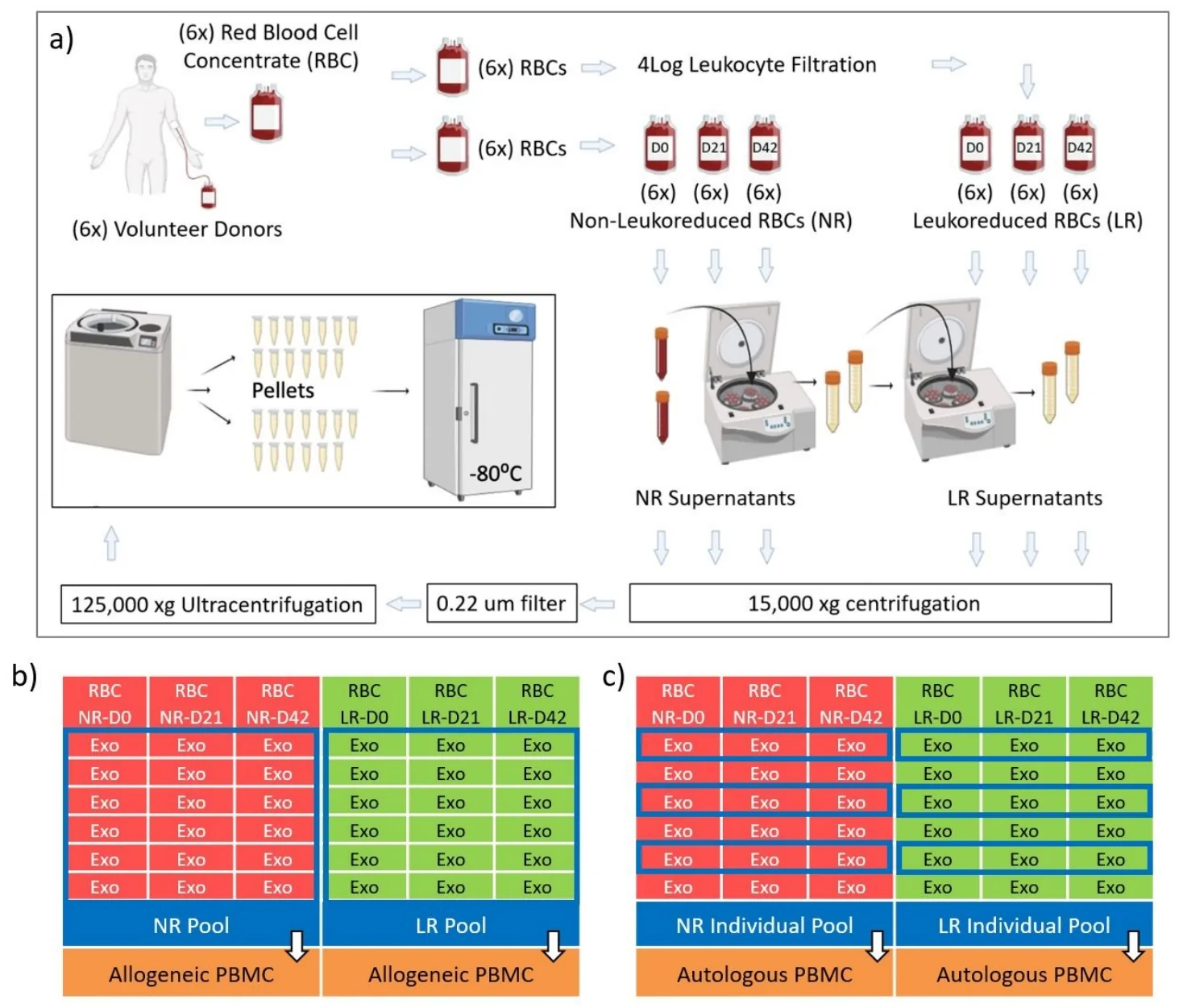
Experimental workflow for the isolation of RBC derived exosomes (RBC-Exos) and establishment of allogeneic and autologous PBMC culture models. (**a**) Exosome isolation procedure. (**b**) Preparation of allogeneic exosome pools. The Allogeneic NR Pool was prepared by mixing all NR-D0-Exo, NR-D21-Exo, and NR-D42-Exo samples. The Allogeneic LR Pool was prepared by mixing all LR-D0-Exo, LR-D21-Exo, and LR-D42-Exo samples in equal concentrations (blue squares). (**c**) Preparation of autologous exosome pools. For autologous NR Individual Pools, NR-D0-Exo, NR-D21-Exo, and NR-D42-Exo samples of each blood donor were mixed in equal concentrations. For autologous LR Individual Pools, LR-D0-Exo, LR-D21-Exo, and LR-D42-Exo samples of each blood donor were mixed in equal concentrations (blue rectangles). Abbreviations: RBC, red blood cell concentrate; NR, non-leukoreduced; LR, leukoreduced; PBMC, peripheral blood mononuclear cell.

**Figure 2:**
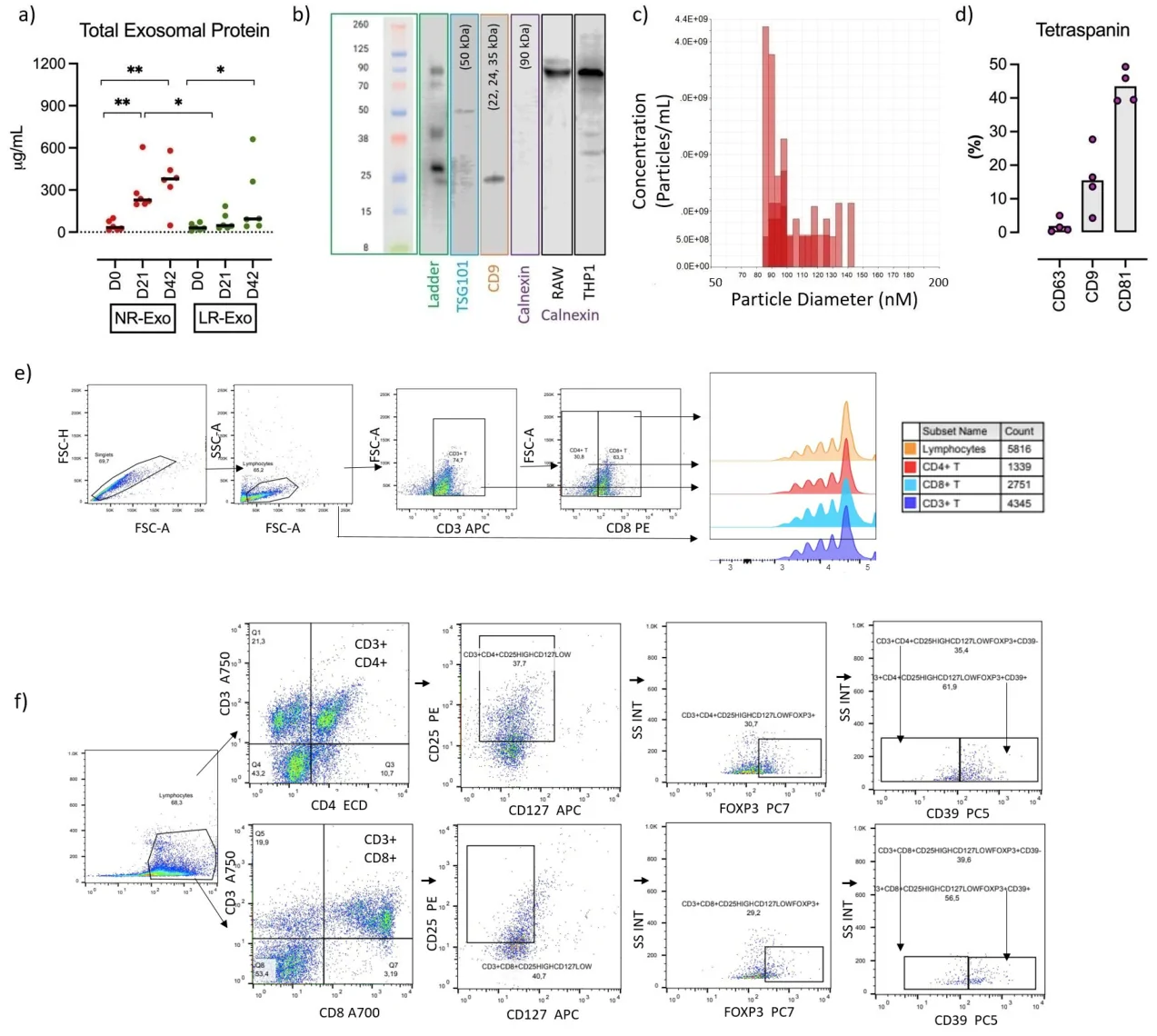
Characterization of exosomes isolated from non-leukoreduced and leukoreduced red blood cell concentrates. (**a**) Total exosomal protein concentrations in NR-Exo and LR-Exo during storage time. * *p* < 0.05 and ** *p* < 0.01. (**b**) Western blot characterization of isolated exosomes. TSG101 and CD9 were detected, whereas calnexin was absent, RAW macrophage lysate and THP-1 cell lysate were included as positive controls for calnexin expression. (**c**) Representative tunable resistive pulse sensing (TRPS) analysis demonstrating the size distribution and concentration of isolated exosomes. (**d**) Flow cytometric characterization of exosome associated tetraspanins. CD9 and CD81 were detected on isolated exosomes. (**e**) Representative gating strategy for proliferation assays. First, single cells were selected using the FSC-A/FSC-H plot, and then the lymphocyte population was defined based on FSC-A/SSC-A characteristics. CD3^+^ T cells within the lymphocytes were identified, and CD4^+^ and CD8^+^ T cells were selected from among the CD3^+^ cells. Cell proliferation was assessed by comparing CFSE fluorescence peaks in the T cell populations. Red indicates CD4^+^ proliferation, blue indicates CD8^+^ proliferation, purple indicates CD3^+^ proliferation, and orange indicates proliferation within the total lymphocyte gate. (**f**) Representative gating strategy for Treg analyses. First, single cells were selected using the FSC-A/FSC-H plot, and then the lymphocyte population was defined based on FSC-A/SSC-A characteristics. CD3^+^ T cells within the lymphocyte gate were gated, and all subpopulation analyses were performed using this gate. CD4^+^ and CD8^+^ T cell subpopulations were selected from within the CD3^+^ cells. The regulatory T cell phenotype was defined as cells expressing CD25HighCD127LowFoxP3High within the CD4^+^ and CD8^+^ T cell populations. To assess CD39 expression, CD39^+^ cells were additionally gated within these regulatory T cell populations. Abbreviations: Exo, exosome; NR, non-leukoreduced; LR, leukoreduced; TRPS, tunable resistive pulse sensing; D0, day 0; D21, day 21; D42, day 42; RAW, RAW 264.7 macrophage cell line; THP-1, human monocytic cell line.

**Figure 3:**
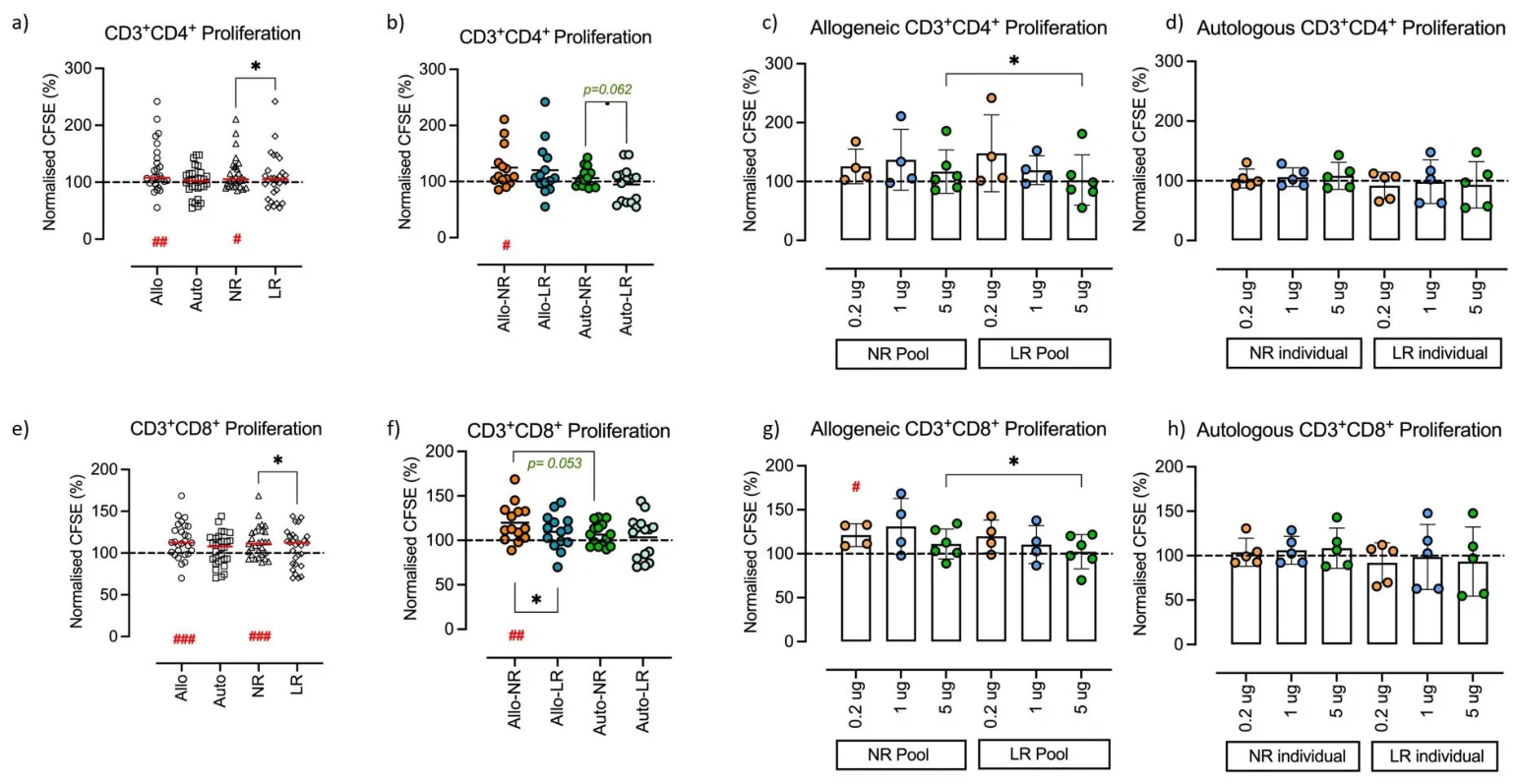
The effects of RBC-derived exosomes (RBC-Exos) on CD4^+^ and CD8^+^ T cell proliferation in allogeneic and autologous PBMC cultures. (**a**) Overall comparison of CD3^+^CD4^+^ T cell proliferation according to culture type (allogeneic vs. autologous) and exosome source (NR-Exo vs. LR-Exo). (**b**) Comparison of CD3^+^CD4^+^ T cell proliferation according to both culture type and leukoreduction status (Allo-NR, Allo-LR, Auto-NR, and Auto-LR). (**c**) The evaluation of effects of pooled exosomes on CD3^+^CD4^+^ T cell proliferation in allogeneic PBMC cultures. (**d**) The evaluation of effects of donor matched exosomes on CD3^+^CD4^+^ T cell proliferation in autologous PBMC cultures. (**e**) Overall comparison of CD3^+^CD8^+^ T cell proliferation according to culture type and exosome source. (**f**) Comparison of CD3^+^CD8^+^ T cell proliferation according to culture type and leukoreduction status. (**g**) The evaluation of effects of pooled exosome on CD3^+^CD8^+^ T cell proliferation in allogeneic PBMC cultures. (**h**) The evaluation of effects of donor matched exosomes on CD3^+^CD8^+^ T cell proliferation in autologous PBMC cultures. Data are presented as individual biological replicates with bars representing the median and interquartile range. The dashed horizontal line indicates the normalized control value (100%). The red line indicates the median values. Statistical significance is indicated as *p* < 0.05. Red symbols (#) indicate significant differences compared with the corresponding control group, whereas black brackets (*) indicate comparisons between experimental groups. # *p* < 0.05, ## *p* < 0.01, ### *p* < 0.001, * *p* < 0.05 and *p* = 0.050–0.065 were defined as trends or limited significance. Abbreviations: Allo, allogeneic; Auto, autologous; NR, non-leukoreduced; LR, leukoreduced; PBMC, peripheral blood mononuclear cell; CFSE, carboxyfluorescein succinimidyl ester; NR Pool, pooled exosomes from non-leukoreduced RBCs; LR Pool, pooled exosomes from leukoreduced RBCs; NR Individual Pool, donor-specific pooled exosomes from non-leukoreduced RBCs; LR Individual Pool, donor-specific pooled exosomes from leukoreduced RBCs.

**Figure 4:**
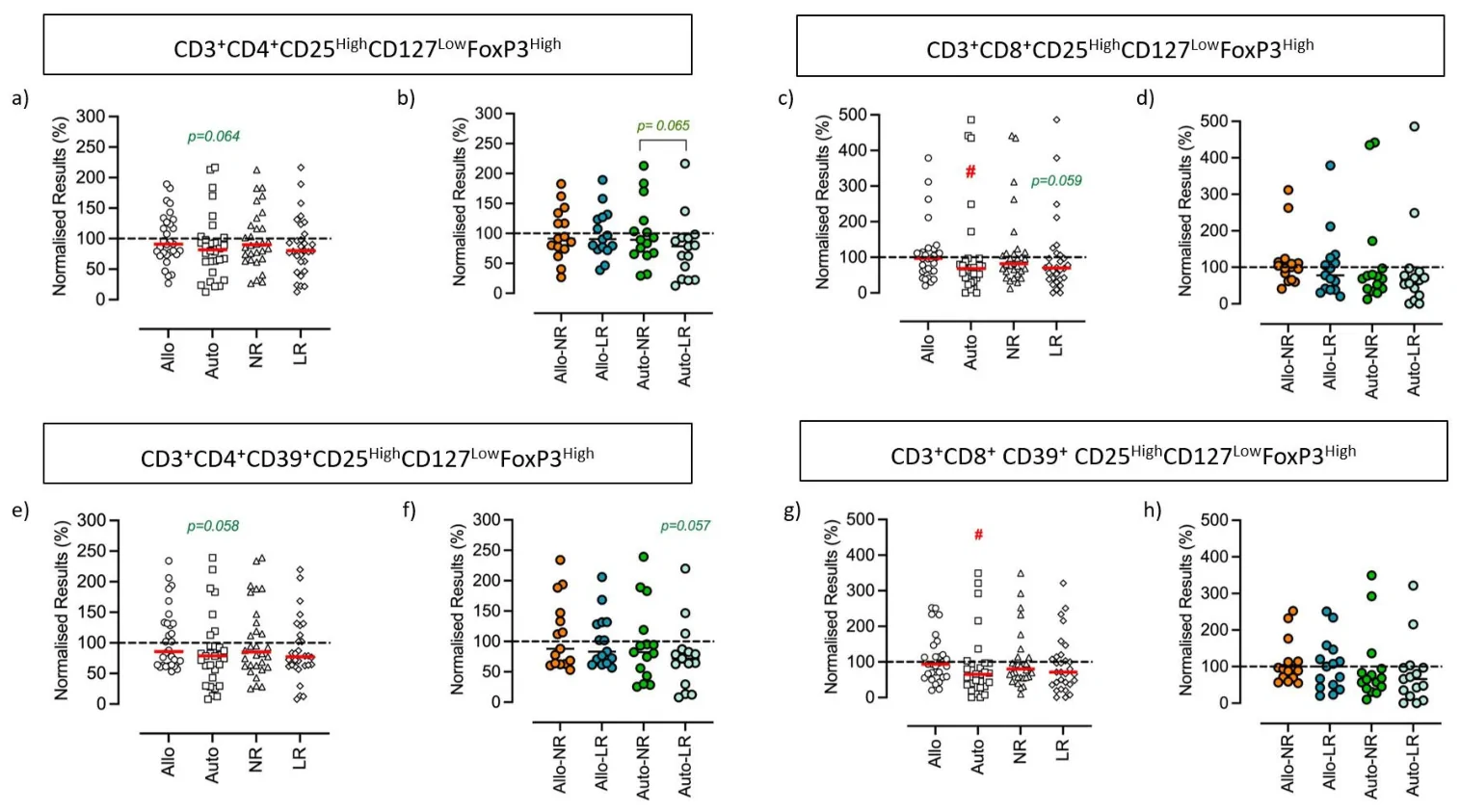
The effects of RBC-Exos on regulatory T-cell populations in allogeneic and autologous PBMC cultures. (**a**) Overall comparison of CD4^+^ Treg cells according to culture type (allogeneic vs. autologous) and exosome source (NR-Exo vs. LR-Exo). (**b**) Comparison of CD3^+^CD4^+^ Treg frequencies according to culture type and leukoreduction status (Allo-NR, Allo-LR, Auto-NR, and Auto-LR). (**c**) Overall comparison of CD8^+^ Treg cells according to culture type and exosome source. (**d**) Comparison of CD3^+^CD8^+^ Treg frequencies according to culture type and leukoreduction status. (**e**) Overall comparison of CD4^+^CD39^+^ Treg cells according to culture type and exosome source. (**f**) Comparison of CD39^+^CD4^+^ Treg frequencies according to culture type and leukoreduction status. (**g**) Overall comparison of CD8^+^CD39^+^ Treg cells according to culture type and exosome source. (**h**) Comparison of CD39^+^CD8^+^ Treg frequencies according to culture type and leukoreduction status. Data are presented as individual biological replicates with horizontal lines representing the median. The dashed horizontal line indicates the normalized control value (100%). The red line indicates the median values. Statistical significance is indicated by *p* < 0.05, while green values denote trends toward significance (*p* ≥ 0.05). Red symbols (**#**) indicate significant differences compared with the corresponding control group. **#** *p* < 0.05, and *p* = 0.050–0.065 were defined as trends or limited significance. Abbreviations: Allo, allogeneic; Auto, autologous; NR, non-leukoreduced; LR, leukoreduced; PBMC, peripheral blood mononuclear cell; Treg, regulatory T cell; FoxP3, forkhead box P3 transcription factor.

**Figure 5:**
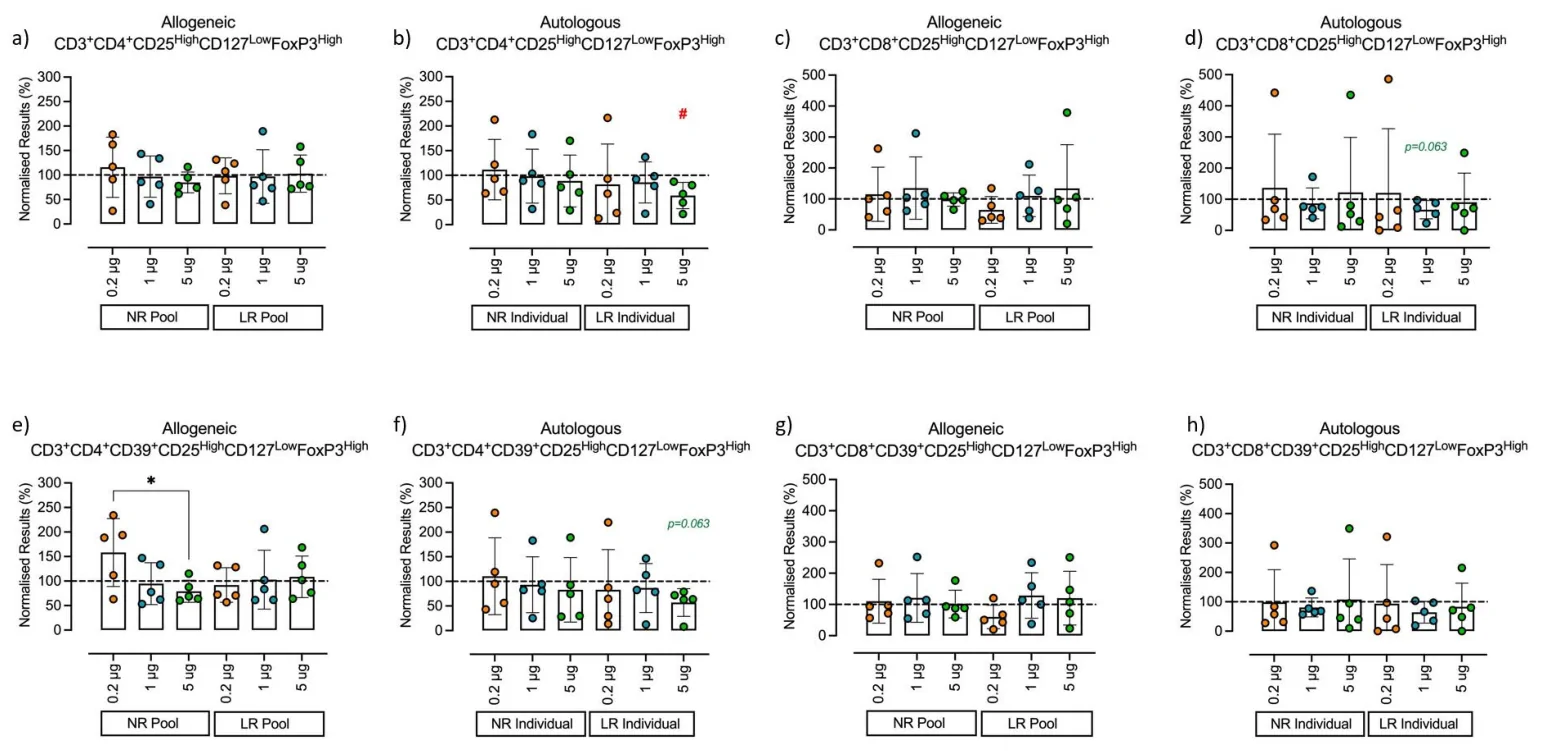
The observed effect of different doses of RBC-derived exosomes on regulatory T-cell populations in allogeneic and autologous PBMC cultures. (**a**) The evaluation of effects of pooled NR and LR exosomes on CD4^+^ Treg cells in allogeneic PBMC cultures. (**b**) The evaluation of effects of donor-specific NR and LR exosomes on CD4^+^ Treg cells in autologous PBMC cultures. (**c**) The evaluation of effects of pooled NR and LR exosomes on CD8^+^ Treg cells in allogeneic PBMC cultures. (**d**) The evaluation of effects of donor-specific NR and LR exosomes on CD8^+^ Treg cells in autologous PBMC cultures. (**e**) The evaluation of effects of pooled NR and LR exosomes on CD4^+^CD39^+^ Treg cells in allogeneic PBMC cultures. (**f**) The evaluation of effects of donor-specific NR and LR exosomes on CD4^+^CD39^+^ T cells in autologous PBMC cultures. (**g**) The evaluation of effects of pooled NR and LR exosomes on CD8^+^CD39^+^ Treg cells in allogeneic PBMC cultures. (**h**) The evaluation of effects of donor-specific NR and LR exosomes on CD8^+^CD39^+^ Treg cells in autologous PBMC cultures. Data are presented as individual biological replicates with bars representing the median and interquartile range. The dashed horizontal line indicates the normalized control value (100%). The red line indicates the median values. Statistical significance is indicated by *p* < 0.05, while green values denote trends toward significance (*p* ≥ 0.05). Red symbols (**#**) indicate significant differences compared with the corresponding control group. **#** *p* < 0.05, * *p* < 0.05, and *p* = 0.050–0.065 were defined as trends or limited significance. Abbreviations: NR, non-leukoreduced; LR, leukoreduced; PBMC, peripheral blood mononuclear cell; Treg, regulatory T cell; FoxP3, forkhead box P3 transcription factor; NR Pool, pooled exosomes from non-leukoreduced RBC; LR Pool, pooled exosomes from leukoreduced RBCs; NR Individual Pool, donor-specific pooled exosomes from non-leukoreduced RBCs; LR Individual Pool, donor-specific pooled exosomes from leukoreduced RBCs.

## Data Availability

The data presented in this study is available on request from the corresponding: author.

## Abbreviations

The following abbreviations are used in this manuscript:

ABT	Allogeneic blood transfusion
D0	Day 0
D21	Day 21
D42	Day 42
Exo	Exosome
FC	Flow-cytometer
FSC	Forward scatter
LR	Leukoreduced
MNC	Mononuclear cell
NR	Non-Leukoreduced
PHA	Phytohemagglutinin
RBC	Red blood cell concentrates
SSC	Side scatter
Tc	CD3^+^CD8^+^ cytotoxic T cells
Th	CD3^+^CD4^+^ helper T cells
Treg	Regulatory T cells
TRIM	Transfusion-related immunomodulation
